# Depletion of intrinsic expression of Interleukin-8 in prostate cancer cells causes cell cycle arrest, spontaneous apoptosis and increases the efficacy of chemotherapeutic drugs

**DOI:** 10.1186/1476-4598-8-57

**Published:** 2009-07-31

**Authors:** Rajendra K Singh, Bal L Lokeshwar

**Affiliations:** 1 Department of Urology and Sylvester Comprehensive Cancer Center, Miller School of Medicine, University of Miami, Miami, Florida, USA; 2 VA Medical Center, Miami, Florida, USA

## Abstract

**Background:**

The progression of all cancers is characterized by increased-cell proliferation and decreased-apoptosis. The androgen-independent prostate cancer (AIPC) is the terminal stage of the disease. Many chemokines and cytokines are suspects to cause this increased tumor cell survival that ultimately leads to resistance to therapy and demise of the host. The AIPC cells, but not androgen-responsive cells, constitutively express abundant amount of the pro-inflammatory chemokine, Interleukin-8 (IL-8). The mechanism of IL-8 mediated survival and therapeutic resistance in AIPC cells is unclear at present. The purpose of this report is to show the pervasive role of IL-8 in malignant progression of androgen-independent prostate cancer (AIPC) and to provide a potential new therapeutic avenue, using RNA interference.

**Results:**

The functional consequence of IL-8 depletion in AIPC cells was investigated by RNA interference in two IL-8 secreting AIPC cell lines, PC-3 and DU145. The non-IL-8 secreting LNCaP and LAPC-4 cells served as controls. Cells were transfected with RISC-free siRNA (control) or validated-pool of IL-8 siRNA. Transfection with 50 nM IL-8 siRNA caused >95% depletion of IL-8 mRNA and >92% decrease in IL-8 protein. This reduction in IL-8 led to cell cycle arrest at G_1_/S boundary and decreases in cell cycle-regulated proteins: Cyclin D1 and Cyclin B1 (both decreased >50%) and inhibition of ERK1/2 activity by >50%. Further, the spontaneous apoptosis was increased by >43% in IL-8 depleted cells, evidenced by increases in caspase-9 activation and cleaved-PARP. IL-8 depletion caused significant decreases in anti-apoptotic proteins, BCL-2, BCL-xL due to decrease in both mRNA and post-translational stability, and increased levels of pro-apoptotic BAX and BAD proteins. More significantly, depletion of intracellular IL-8 increased the cytotoxic activity of multiple chemotherapeutic drugs. Specifically, the cytotoxicity of Docetaxel, Staurosporine and Rapamycin increased significantly (>40% at IC_50 _dose) in IL-8 depleted cells as compared to that in C-siRNA transfected cells.

**Conclusion:**

These results show the pervasive role of IL-8 in promoting tumor cell survival, and resistance to cytotoxic drugs, regardless of the cytotoxic mechanism of antiproliferative drugs, and point to potential therapeutic significance of IL-8 depletion in men with AIPC.

## Background

Prostate cancer ranks second in incidence and mortality among all cancers in men in the United States [1]. The castration resistant, androgen-independent prostate cancer (AIPC) accounts for most mortalities from this disease. The AIPC is also associated with poor response to chemotherapy drugs, and therefore, high mortality with an estimated life span of 2–4 years [2,3]. Many factors contribute to this state of the disease, including multiple survival mechanisms, resistance to apoptosis and development of resistance to therapeutic drugs. The present investigation is to understand whether these are contributed by the ability of AIPC cells to chemokines such as Interleukin-8 (IL-8) in a paracrine or autocrine fashion.

IL-8 is a multifunctional chemokine, involved in inflammation mediated neutrophil infiltration and chemotaxis [4]. A member of the Cysteine-X-Cysteine (CXC) motif chemokines, IL-8 is one of the most promiscuous mediators of immune and cellular functions, including motility, invasion and activation of survival and proliferative pathways in cells of mesenchymal lineage and in aggressive tumor cells [5,6]. The up-regulation of IL-8 in various pathologies is attributed to the structure of IL-8 promoter. The IL-8 promoter binds to Nuclear Factor kappa B (NF-kB), AP-1 and other inflammation related enhancers [7]. Expression of IL-8 in tumor cells may also be associated with constitutive activation of inflammatory pathway, such as that initiated by activation of NF-kB, AP-1 and hypoxia inducible factor-1α in some tumor cells [8,9]. Since IL-8 is a secreted protein, conditions prevailing in the tumor microenvironment, such as infiltrating monocytes and lymphocytes, may further increase the influence of IL-8 in such tumors [10-12].

IL-8 binds to two cell surface G-protein coupled receptors (GPCR), IL-8 receptor A and IL-8 receptor B or CXCR1 and CXCR2, respectively. IL-8 receptors, unlike IL-8, are constitutively expressed in both mesenchymal and epithelial cells [13] and CXCR2 binds multiple other ligands [14,15]. IL-8 induced cellular functions are mediated through the activation of these two receptors. Studies have shown that disease progression and metastasis may be associated with over-expression of IL-8 [6,16]. However, the mechanism by which IL-8 promotes various pro-survival and anti-apoptotic functions is unclear at present.

AIPC cells synthesize and secrete IL-8 but not normal prostatic epithelial cells, primary tumors and in androgen-dependent/responsive prostate cancer cell lines [17,18]. Several investigators have reported the generation of androgen-independent cell clones that are not only capable of growing in the absence of androgen, but also secrete IL-8 [19,20]. We reported previously, that forced expression of IL-8 in androgen-responsive cells leads to androgen-independent cell growth and up regulation of several key attributes of invasion and metastasis [21]. In addition, over expression of IL-8 in IL-8 secreting AIPC cells causes them to grow as more aggressive and angiogenic tumors in vivo [22,23]. However, the mechanism of growth advantage rendered by constitutive IL-8 production, without over expression, is not delineated. For example, earlier studies attributed most of the tumor growth promoting activities of IL-8 to its effect on angiogenesis, not the survival [22,23]. We hypothesized that IL-8 is a survival factor that not only promotes proliferation pathway, but also controls apoptotic pathway, due to its interaction with protein kinase-B (AKT) and NF-kB. The focus of the present report is to demonstrate the contribution of IL-8 in prostate cancer cell-survival, invasion and resistance to chemotherapeutic drugs in two AIPC cell lines, PC-3 and DU145 by RNA interference.

## Results

### Effect of siRNA directed IL-8 silencing in AIPC cells

**Figure 1:**
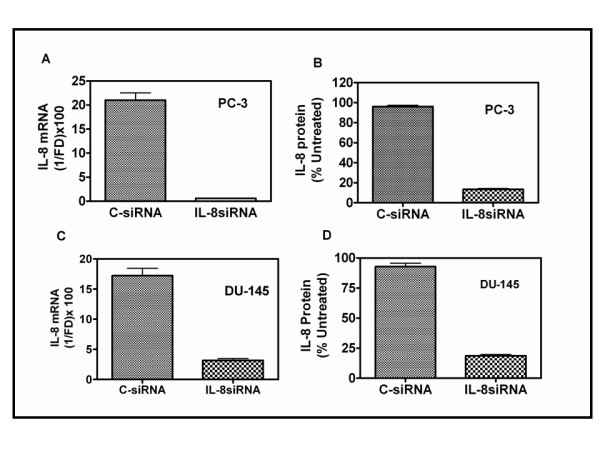
**Depletion of IL-8 in PC-3 and DU145 cells by siRNA transfection**: **A and C: Decrease in IL-8 mRNA levels**: Cells were transfected with 50 nM of control-siRNA (C-siRNA) or Smartpool siRNA against IL-8 (IL-8 siRNA). Total RNA was extracted 48 h after transfection to determine the levels of IL-8 mRNA by Q-RT PCR, as described in Materials and Methods (text). Each RNA sample was also simultaneously subjected to Q-RT PCR to determine the level of GAPDH mRNA for normalization. The threshold cycle (Ct) of each reaction generated by the iCycler program (Bio-Rad Inc.) was used to determine the relative mRNA levels in the test samples, using the formula: Fold Difference (FD) = 1-(2^ΔCt^), where, ΔCt = (Ct_test RNA _- Ct_GAPDHRNA_). Final value of IL-8 mRNA, relative to that of GAPDH was expressed as: 1/FD × 100 to obtain integer values, in arbitrary units. **B and D**: **Levels of IL-8 protein secreted into the culture medium**, 48 h after transfection. IL-8 levels were measured using an ELISA kit. Levels are expressed as % of untreated control. Untreated PC-3 cells secreted ~ 60 ng IL-8/10^6 ^PC-3 cells/24 h and ~ 10 ng IL-8/10^6 ^DU145 cells/24 h.

Transfection of PC-3 and DU145 cells with Smartpool siRNA, directed against IL-8 mRNA reduced the expression of IL-8 mRNA in a dose-dependent manner (50–95%) at a concentration range of 25 nM to 100 nM. However, non-target, scrambled sequence-siRNA (C-siRNA) and IL-8 siRNA, both, showed off-target toxicity at ≥ 100 nM. At 50 nM, C-siRNA did not cause an alteration in cell viability, or IL-8 mRNA, but transfection with 50 nM IL-8 siRNA caused 98% and 92% decrease in IL-8 mRNA levels in PC-3 and DU145 cells, respectively (Fig. 1A &1C). Furthermore, we observed mean decreases of 92% and 85% in secreted IL-8 levels at 72 h after transfection in PC-3 and DU-145 cells, respectively (Fig. 1B &1D).

### IL-8 depletion causes inhibition of PC-3 and DU145 cells proliferation

**Figure 2:**
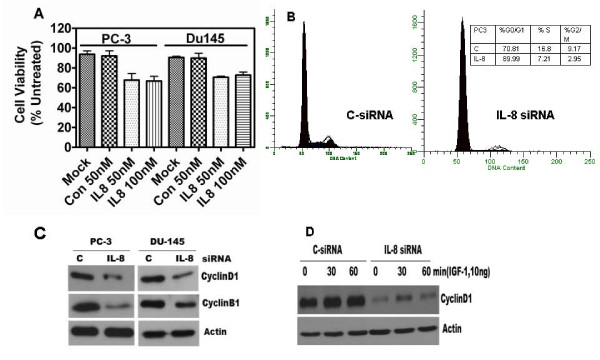
**IL-8 depletion in AIPC cells causes decrease in cell proliferation and cell cycle arrest**: **(A)**. Cell viability was measured using the MTT assay, 48 h after transfection. Triplicate wells were used for each measurement, experiment repeated three times per cell line. Error bars: mean ± s.e.m, n = 3. (**B**). **Cell cycle arrest at G1/S boundary**. Percent of cells in each of the three cell cycle phase was determined by flow cytometry, 48 h after siRNA transfection. Results shown are from one transfection experiment, similar results were obtained in two separate determinations. (**C**). **Levels of Cyclin D1 and Cyclin B1 expression**: Detected by immunoblotting, as described in the text. β-actin levels, as shown, were used to normalize the protein levels of Cyclin D1 and Cyclin B1. The decrease in the levels of Cyclin D1 by IL-8 depletion was 68% in PC-3 and 52% in DU145 cells. The decrease in Cyclin B1 level was 73% in PC-3 and 41% in DU145, as determined by densitometry measurement of band intensities and normalized to β-actin levels. **D. IL-8 siRNA transfection decreases growth factor (IGF-1) mediated mitogenic stimulation**. Cyclin D1 in cell lysates, prepared from PC-3 cells stimulated with IGF-1, was detected by western blotting. Levels of Cyclin D1 were normalized to β-actin (Actin) levels detected by re-probing the same blots.

Reduction of IL-8 expression by transfection with 50 nM IL-8 siRNA caused a significant decrease in cell viability. Decrease in cell viability was measured with an MTT reduction assay, 48 h following transfection. Cell viability decreased by 30 ± 5.2% in PC-3 and 28 ± 4.7% in DU145 cells, respectively, to that of mock transfection. As shown in Fig. 2A, C-siRNA had no significant effect on cell proliferation in either cell lines. Furthermore, IL-8 siRNA had no effect on the proliferation of androgen-responsive, IL-8 non-secreting LNCaP cells (data not shown). Since siRNA-mediated gene silencing lasts two to four days in cell culture, we did not use clonogenic survival assays (colony assay) or generate cell growth curves to determine the long-term growth inhibition. To distinguish between alteration in cell proliferation and cell viability, we used cell cycle-phase fractionation by flow cytometry, which provided more analytical details of cell cycle distribution of transfected cell cultures.

### IL-8 depletion affects the cell cycle distribution

As shown in Fig. 2B, IL-8 depletion by siRNA transfection caused an arrest of PC-3 cells in G1 phase of cell cycle, and prevented their entry into S-and G2/M phase. The fraction of cells in G0/G1 phase in IL-8 siRNA transfected cells was significantly higher (85%) compared to that of C-siRNA transfectants (68%) (Table insert in Fig. 2B). Similar cell cycle phase analysis in DU145 cells also showed the G1 phase arrest when transfected with IL-8 siRNA (data not shown).

We next analyzed the levels of key molecules that control the progression of cells from G1 to S-phase of the cell cycle. The expression of Cyclin D1 and Cyclin B1 decreased significantly in IL-8 depleted cells. We detected decreased levels of Cyclin D1 and Cyclin B1 in both PC-3 and DU145 cells (Fig. 2C). Furthermore, growth factor induced increase in Cyclin D1 level was modest in IL-8 depleted cells, when compared to those with C-siRNA transfected cells (Fig. 2D). As shown in Fig. 2D, external addition of IGF-1 increased Cyclin D1 level by ~50% in C-siRNA transfectants within 30 min and stayed high for 60 min, however, it increased only ~30% in IL-8siRNA transfected cells. However, in contrast to IL-8 siRNA transfectants, C-siRNA transfectants showed increased Cyclin D1 expression after IGF-1 addition.

### External addition of IL-8 rescues IL-8 siRNA mediated growth arrest

**Figure 3:**
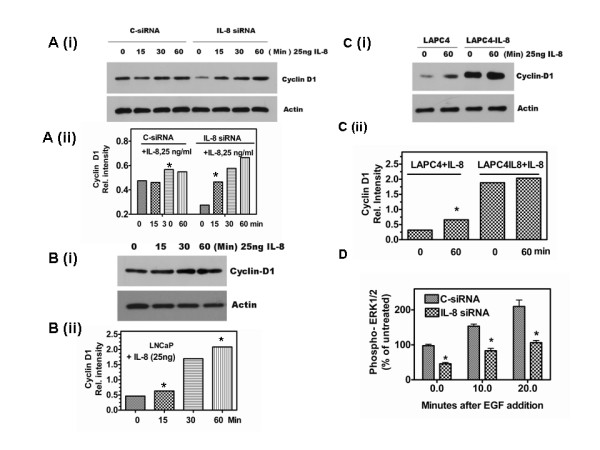
**External IL-8 restores the functions of IL-8 depleted by siRNA transfection**: Effect of addition of recombinant human IL-8 (rhIL-8) on Cyclin D1 level was determined by western blotting. PC-3 cells transfected with 50 nM C-siRNA or IL-8 siRNA and cultured in complete medium for 48 h were stimulated with rhIL-8 (25 ng/ml) and harvested by SDS-sample buffer at 0, 15, 30 and 60 min, and probed for Cyclin D1 and β-actin (A (i)). **Expression of Cyclin D1 relative to β-actin in PC-3 cells**: Cyclin D1 was quantified by densitometry and expressed as a ratio of the band intensity with respect to β-actin (A (ii)). Note that Cyclin D1 level rose significantly in cultures depleted with IL-8 but supplemented with external IL-8. **B. IL-8 stimulates Cyclin D1 in LNCaP cells**. LNCaP cells cultured in complete medium for 48 h were stimulated with rhIL-8 before harvesting and determining Cyclin D1 level, as described for A. **C**. **Cyclin D1 in IL-8 stimulated LAPC4 and LAPC4IL-8 cells**. (i). The experimental conditions to determine Cyclin D1 were identical to the one used in B, except that LAPC4IL-8 cells were used that constitutively produce IL-8 (8 ng/10^6 ^cells/24 h) [18]. Note that, while significant increase in Cylcin D1 is observed in LAPC-4 cultures stimulated with IL-8, the increase in Cyclin D1 level was negligible in LAPC4-IL-8 cells (C(ii)). **D. Phosphorylated ERK1/2 levels are lower in IL-8 depleted cells: Levels were **determined by an ELISA from cell lysates prepared from EGF-stimulated PC-3 cells with C-siRNA or IL-8 siRNA transfection. Error bars indicated are Mean ± SD from pooled data of two independent determination with triplicate samples. Significance of the observation was tested using Student's t-test as described in Methods. (* indicates p = 0.05, determined using t-test as described in the Methods.).

Using levels of Cyclin D1 as readout, we examined whether IL-8siRNA mediated growth arrest is specific to IL-8 depletion or due to events unrelated to IL-8. Since external addition of IL-8 up regulates Cyclin D1 in PC-3 cells by increasing its translation [24], we examined whether such treatment rescues siRNA transfected cells. We treated C-siRNA or IL-8siRNA transfected PC-3 cells with IL-8 (25 ng/ml) for up to one hour and determined the level of Cyclin D1 by western blotting. As shown in Fig. 3A, external addition of IL-8 in C-siRNA transfected PC-3 cells (in which cells continue to make IL-8) did not induce significant increase in Cyclin D1. However, external addition of IL-8, to a PC-3 cell cultures at 48 h after transfecting with IL-8 siRNA increased the Cyclin D1 level significantly, in a time-dependent manner (Fig. 3A(ii)). In addition, we observed that external addition of IL-8 increases Cyclin D1 level in cells that do not constitutively produce IL-8, such as LNCaP (Fig. 3B(i) and 3B(ii)) and LAPC-4 (Fig. 3C(i)). These results corroborate the specificity of IL-8 siRNA and both autocrine and paracrine function of IL-8 in stimulating cell cycle progression via Cyclin D1 accumulation. The observation that elevated Cyclin D1 level in IL-8 producing PC-3 cells but lack of stimulation of Cyclin D1 translation following external IL-8 addition in these cells prompted us to inquire whether constitutive induction of intracellular IL-8 by forced expression renders these cells insensitive to paracrine stimulation with IL-8. Indeed, as shown in Fig. 3C(i, ii) we found high level of Cyclin D1 in LAPC4-IL-8 cells that constitutively produce IL-8 due to constitutive expression of IL-8sense cDNA transfection, as described before [21].

Next, we investigated whether IL-8 depletion alters the mitogenic signaling cascade. Specifically, we determined whether IL-8 depletion leads to an inhibition or attenuation of MAP kinases, such as ERK1/2. MAP kinase activity in IL-8 depleted cells was about 50% of the C-siRNA transfected cells, however, following addition of EGF (20 nM) there was a rapid increase in MAP kinase activity in both C-siRNA and IL-8 siRNA transfected cells (Fig. 3D). Although, the rate of increase in IL-8 siRNA transfected cells was comparable to that of C-siRNA transfected cells, the absolute level was only ~40% of that of C-siRNA transfectants. These results demonstrate that IL-8 depletion can potentially cause attenuation of growth factor signaling in tumor tissue.

### IL-8 siRNA down regulates key factors that control survival and metastatic pathway

**Figure 4:**
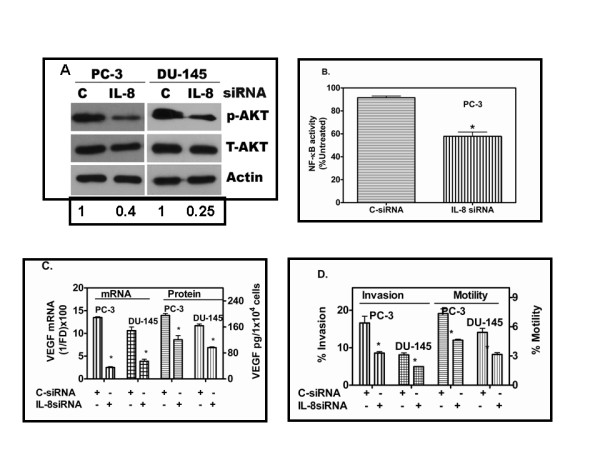
**Decrease in survival and angiogenic factors in IL-8 depleted AIPC cells**: **(A)**. IL-8 depletion reduced the steady state levels of activated AKT (Phospho-AKT). Phospho-AKT levels were detected by western blotting in cell lysates, prepared 48 h following C-siRNA or IL-8 siRNA transfection of PC-3 or DU145 cells. Numbers at the bottom of the gel indicate p-AKT band intensity relative to that of C-siRNA transfectants, after normalizing the band intensities to that of T-AKT. **(B). NFkB activity in PC-3 cells **measured using a NFkB-Luciferase reporter construct co-transfected with control or IL-8 siRNA and TK renilla plasmid as described in the text. **(C). VEGF levels in IL-8siRNA or C-siRNA transfected PC-3 and DU145 cells**. Relative levels of VEGF mRNA, 48 h after transfection, were determined by Q-RTPCR. VEGF protein levels in culture-conditioned medium of transfected cells from 24 h-48 h, following transfection, were determined using an ELISA kit (R&D system Inc.) * p < 0.05. **D**. **Decrease in the invasion and motility activity of PC-3 and DU145 cells transfected with IL-8 siRNA**. Fractions of cells migrated to bottom wells either due to chemotaxis or invaded the Matrigel-coated filters were determined by MTT assay, as described in the text. The data shown is from a single experiment with triplicate wells, similar results were obtained from two independent experiments. Note the %-invaded cells is higher than that of % motility because, the invasion assay was terminated at 48 h and motility assay at 24 h. At 24 h, MTT assay was too insensitive to estimate of %-invaded cells in DU145 cells. Error bars: Mean ± SD. * represents the probability (p) that the measured values presented from IL-8siRNA and C-siRNA transfectants are the same; p < 0.001 in all experiments (B-D).

We examined two key factors that are involved in survival and metastatic pathway, protein kinase B (AKT) and NF-kB activities [25,26]. As shown in Fig. 4A, we observed a significant reduction in phosphorylated form of AKT in IL-8 depleted cells as compared to the cells transfected with C-siRNA alone. The decrease in phospho-AKT to total AKT was more than 2-fold in IL-8siRNA transfectants. Phospho-AKT level was decreased by 60% in PC-3 cells and 75% in DU145 cells transfected with IL-8 siRNA (Fig. 4A). Furthermore, we found a significant decrease (38.5% ± 4.8%) in the endogenous NFkB activity in IL-8 depleted cells (Fig. 4B), assayed using an NF-kB reporter construct [21].

### IL-8 depletion reduced VEGF expression

Several investigators have reported a close link between tumor angiogenesis and IL-8 [[10,11,22], and [23]]. Since IL-8 and VEGF are implicated in increasing angiogenic potential in PC-3 cells [23], we investigated whether IL-8 depletion reduces the expression of angiogenic factors, such as the VEGF. As shown in Fig. 4C, IL-8 depletion by siRNA transfection significantly reduced both mRNA (70%) and protein (<33%) levels of VEGF in both PC-3 and DU145 cells transfected with IL-8siRNA.

### IL-8 depletion causes a decrease in tumor cell chemotactic motility and chemo-invasive potential

IL-8 affects both motility and invasive potential when added externally at high concentration (25 nM) [21], the role of autocrine IL-8 in tumor cell motility and invasive potential in prostate cancer is not been reported until now. More importantly, although several studies have demonstrated its endocrine/paracrine activities, whether autocrine IL-8 signaling (i.e., signaling due to the intracellular production of IL-8), is sufficient to cause significant motility and invasive activity, the two critical determinants of metastatic phenotype is not tested until now. As illustrated in Fig. 4D, IL-8 depleted cells showed a significant decrease in both chemotactic motility and chemoinvasion. The decrease in chemotactic motility in PC-3 cell toward 10% fetal bovine serum (40% ± 5.5%) was comparable to that of chemo-invasive activity (44.7% ± 3.7%). In DU145 cells, decreases of 36.3% ± 2.7% invasion versus 42.7% ± 4.4% in motility was observed when transfected with IL-8 siRNA.

### Enhancement of apoptosis with endogenous IL-8 depletion

**Figure 5:**
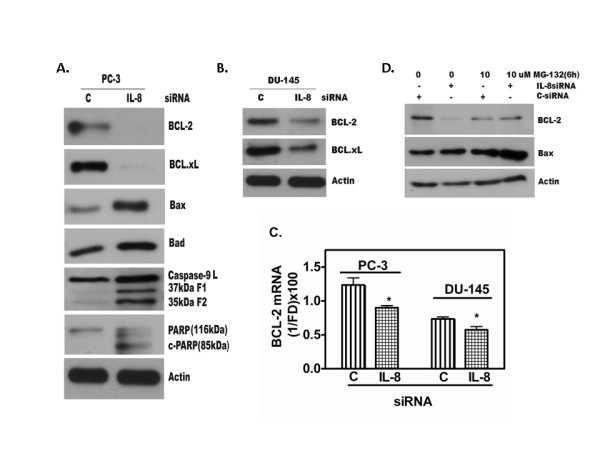
**IL-8 depletion causes decrease in anti-apoptotic proteins and increase in pro-apoptotic proteins in AIPC cells**. **A. Depletion of IL-8 in PC-3 **and (**B**) **DU145 cells **caused increased spontaneous apoptosis detected by caspase-9 activation; cleaved caspase-9 fragments, both of and 37 kDa and 35 kDa along with 39 kDa latent caspase-9 and cleaved poly(ADP-ribose)polymerase (PARP) (85 kDa) proteins are shown. Levels of BCL-2, BCL-xL, BAX and BAD were detected by western blotting of cell lysates prepared 48 h after transfecting the cells with 50 nM IL-8 siRNA or C-siRNA. β-Actin level is shown to ascertain equal protein loading in all lanes. **C. Decrease in BCL-2 level **was due to decrease in mRNA level determined by Q-PCR. **D. The decrease in BCL-2 and increase in BAX level **may also be due to rapid degradation of BCL-2 protein that in turn resulted in increased level of BAX. Inhibition of proteasome degradation of BCL-2 by MG132 shows unchanged level of BCL-2 between C-siRNA transfected control and IL-8siRNA transfected cells (Lanes 3 and 4).

Since we found IL-8 depletion decreases cell survival, we investigated whether this is due to an increase in spontaneous apoptosis following siRNA transfection. The cell lysates of PC-3 and DU145 cells, prepared 48 h after transfection with IL-8 siRNA or C-siRNA, were analyzed for apoptosis markers by western blotting. We analyzed the levels (85-kDA fragment) of cleaved Poly-(ADP Ribose) polymerase (PARP) protein [27]. PARP is cleaved by activated caspase-3 [28]. Caspase-3 is cleaved by Caspase-9 due to mitochondrial permeability increase and the release of Cytochrome C [29]. Since cleaved PARP is the signature event in apoptosis, we rationalized that analysis of cleaved PARP level should indicate spontaneous apoptosis in IL-8 siRNA transfected cells. Indeed, IL-8 siRNA transfected cells showed increased Caspase-9 activity and increased PARP cleavage (Fig. 5A). These experiments suggest that in IL-8 expressing cells, IL-8 may be suppressing spontaneous apoptosis, by yet unknown mechanism. In addition, these events are also linked to the levels of BCL-2, BCL-xL, BAX and BAD proteins [30]. As shown in Fig. 5A, we found significant increase in both caspase-9 activation and increased PARP levels in IL-8 siRNA transfectants when assayed 48 h after transfection.

### IL-8 depletion causes alteration in apoptosis-related proteins

Earlier reports have shown that apoptosis suppressor proteins, BCL-2 and BCL-xL are constitutively higher in IL-8 expressing PC-3 and DU145 cells, compared to that in IL-8 non-secreting LNCaP or LAPC4 cells [30]. As shown in Fig. 5A and Fig. 5B, western blot analysis showed that the transient transfection with IL-8 siRNA resulted in significant reduction of BCL-2 protein 48 h after transfection. Consistent with this finding in PC-3 cells, we observed similar results in DU145 cells after IL8-siRNA transfection (Fig. 5B). We noticed significant reduction of BCL-2 in DU145 cells transfected with IL8 siRNA compared to that of C-siRNA. We further analyzed the BCL-xL protein expression in IL-8 siRNA and C-siRNA transfectants of PC-3. As shown in Fig. 4A &4B, we were unable to detect BCL-xL expression in PC-3 cells transfected with IL-8 siRNA, although in similarly transfected DU145 cells expressed a detectable level of BCL-xL protein.

We further tested whether reduction of BCL-2 and BCL-xL protein expression changed the proportion of pro-apoptotic BAX-BAD proteins [31-33]. We used the western blotting to compare the levels of these proteins in cell lysates of IL-8 siRNA and C-siRNA transfected cultures. As compared to C-siRNA, IL-8 siRNA transfectants showed significantly increased BAX and BAD proteins (Fig. 5A and 5B).

We analyzed whether the down-regulation of apoptosis suppressor protein in AIPC cells is due to decrease transcription or protein turnover (ubiquitylation and proteasomal degradation), or both. We performed Q-RTPCR analysis of BCL-2 mRNA expression and protein turnover analysis using 26S-proteosome inhibitor, Carbobenzoxy-L-leucyl-L-leucyl-L-leucinal Z-LLL-CHO (MG132, 10 μM) [21]. As shown in Fig. 5C, we found a steep decline in BCL-2 mRNA level in IL-8 siRNA transfected cells, compared to the C-siRNA transfected cells. Treatment of C-siRNA and IL-8 siRNA transfected cells with MG132, for 6 h after 40 h following transfection, showed a slight decrease in BCL-2 levels in MG132 treated samples, in both control and IL-8 siRNA treated samples, indicating the toxicity of MG132 in PC-3 cells (Fig. 5D). However, MG132 treated samples retained higher level of BCL-2 in IL-8 depleted cultures, compared to that in IL-8 siRNA transfected cells, without incubation with MG132. These results show that despite the toxicity of MG132 in PC-3 cells, IL-8 depletion causes further decrease in BCL-2 protein. Thus, IL-8 is likely to participate in both transcription and translational control of BCL-2. Compared to the near-total decline in BCL-2 level in IL-8 depleted cells, it caused, not only an increase in BAX mRNA (data not shown), but also showed a significant increase in protein levels (Fig. 5A).

### IL-8 depletion in AIPC cells increases the chemosensitivity to anticancer drugs

**Figure 6:**
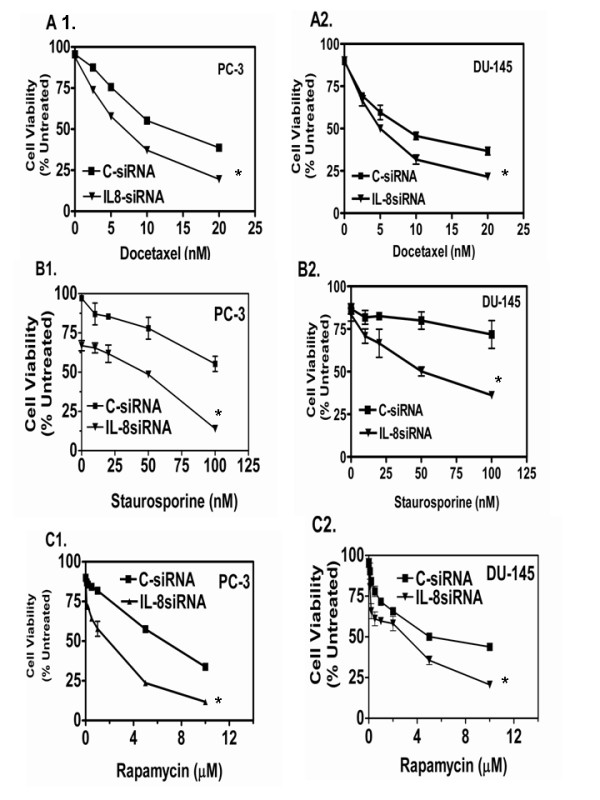
**IL-8 depletion increases the cytotoxicity of chemotherapeutic drugs in AIPC cells**. IL-8 siRNA or CsiRNA transfected PC-3 and DU145 cells were exposed to cytotoxic drugs, 24 h after transfection. Cell viability was evaluated 48 h later (72 h post-transfection) by MTT reduction assay. Cytotoxicity of all three drugs, Docetaxel, Staurosporine and Rapamycin were equally increased in both DU145 and PC-3 cells, indicating chemo-sensitization was not likely depended on the mechanism of cytotoxic drug activity but due to alteration in cell survival mechanism caused by IL-8 depletion. Results shown are Mean ± SEM from three independent experiments, normalized with respect to samples with or without drug treatment. Note: All cytotoxicity data are normalized with respect to the reduction in cell viability due to IL-8siRNA transfection. The cytotoxicity was significantly higher (p < 0.04) at all concentrations of Docetaxel, and Rapamycin in PC-3 and DU145 cells, with Staurosporine in DU145 cells and at highest concentration tested in for all drugs in both cell lines. We analyzed the cytotoxicity data by non-linear regression and tested the mean survival values by t-test.

Since IL-8 depletion decreases the activity of NF-kB, AKT, BCL-2 and BCL-XL, we investigated whether this also affects response to cytotoxic, anticancer drugs. We chose docetaxel, an inhibitor of microtubule depolymerization that blocks cells at G2/M phase [34], staurosporine, a strong inhibitor of protein kinase C and apoptosis inducing drug [35] and rapamycin, an S6-kinase inhibitor [36]. We chose these drugs as representative chemotherapeutic drugs each with unique mechanism of action in tumor cells. The cell cultures transfected with C-siRNA or IL-8 siRNA for 24 h were exposed to several concentrations of each drug for the next 48 h. Cell viability was estimated in untreated control, single treatment alone, and combined siRNA and drug exposed cultures by MTT assay. The combination of 10 nM of docetaxel and IL-8 siRNA transfection significantly enhanced cytotoxicity in PC-3 cells. We found their survival decreased to <10% (>90% decrease in viability) when the cultures were exposed to docetaxel 24 h after IL-8 siRNA transfection, as compared to the 28% survival with docetaxel plus C-siRNA transfection-combination (Fig. 6A). Similarly, as illustrated in Fig. 6B, cell viability of IL-8siRNA transfected cultures, treated with 100 nM Staurosporine, was <10% compared to >50% viability of cultures transfected only with C-siRNA, indicating a 40% increase in cytotoxicity due to IL-8 knockdown. We obtained similar results in DU145 cells treated with the staurosporine and siRNA (Fig. 6, right panels). Further, a significant reduction in viability also was observed in the IL-8 siRNA transfectants treated with rapamycin. We found >90% reduction in viability in IL-8 siRNA transfected cultures treated with rapamycin compared to <45% reduction in cell viability of C-siRNA transfected cells treated with rapamycin. The IL-8 siRNA and C-siRNA transfectants of DU145 cells treated with rapamycin (10 μM) for 24 hr, showed cell viability of <20% and >45%, respectively.

## Discussion

This study demonstrated the capability of siRNA in silencing IL-8 mediated autocrine-regulation of major functions of AIPC cells. We observed that depletion of endogenous expression of IL-8 (over 90%) by siRNA decreased PC-3 and DU145 cell proliferation, cell cycle progression, angiogenic potential and up-regulated spontaneous apoptosis. Furthermore, since its depletion reduced the levels of Cyclin D1 and Cyclin B significantly, we also provide the evidence that endogenous IL-8 stimulates Cyclin D1 synthesis with or without a mitogenic factor, such as IGF-1 or exogenous IL-8 stimulation, (Fig. 2B). In addition to the decrease in Cyclin D1 levels, we also observed a steep decrease in the level of other nuclear proteins involved in cell cycle progression, such as Cdk2 (data not shown), and Cyclin B1. The phenotype of this inhibition is seen as the cell cycle arrest at G1 to S-phase transition.

This work complements and extends the previous work [22,23]. In earlier report, it was shown that anti-sense cDNA mediated silencing of IL-8 in PC-3M and PC-3M-LN4 cells, two highly metastatic variants of PC-3, caused a reduction in tumorigenicity, angiogenesis and metastasis [22]. The authors reported a 5–10 fold reduction in IL-8 mRNA and protein levels in cell culture studies, and ~50% reduction in IL-8 in tumors. This compares to our finding that siRNA mediated silencing resulted in >98% reduction in IL-8 mRNA and 91% reduction in IL-8 protein in vitro, which led to dramatic changes in the cellular phenotype. Whether this reduction leads to similar anti-tumor activity *in vivo *is not tested at present, since siRNA mediated gene silencing is transient and unsuitable, at present, for testing its efficacy *in vivo *on tumor growth.

MacManus CF et al., [24] reported that external addition of IL-8 regulates Cyclin D1 synthesis at the translation stage via S6 kinase-mediated ribosomal phosphorylation mechanism. In addition, they also showed external addition of IL-8 causes AKT phosphorylation and activation of mTOR pathway in PC-3 cells. As we have shown in this report and that of others, PC-3 cells constitutively produce significant amount of IL-8. Therefore, extracellular exposure to IL-8 may not be necessary to elicit some of the IL-8 mediated signaling. We found that exogenous addition of IL-8 only moderately up regulated Cyclin D1 in both PC-3 cells (Fig. 3A). However, in IL-8 depleted PC-3 cells and in those cells that do not constitutively produce IL-8 (e.g., LNCaP and LAPC-4), external addition of IL-8 significantly up regulated Cyclin D1 (Fig. 3A, B, and 3C). Thus, IL-8 is capable of inducing cell proliferation in both IL-8 non-producing androgen responsive CaP cells and in AIPC cells, either by endocrine/paracrine, or by autocrine mechanism.

PC-3 cells form rapidly growing tumors in mice, without any external stimulation by IL-8. Autocrine secretion in the only mechanism by which IL-8 is available to tumor cells in xenografts. The mouse homolog of IL-8 has poor affinity to IL-8 receptors, CXCR1 and CXCR2 in human cells, although human IL-8 binds to murine IL-8 receptors, which may be a cause of increased angiogenesis activity in xenografted PC-3 tumors [37]. However, we do find that mitogenic signaling by IL-8 is mediated by autocrine signaling, through binding to the cell surface receptors of IL-8, CXCR1 and CXCR2. Interestingly, as shown in Fig. 2D, mitogenic signaling by other growth factor, such as IGF-1 also is attenuated without the endogenous expression of IL-8, suggesting IL-8 activated intracellular signaling may synergistically enhance other MAP kinase-induced signals. Indeed, IL-8 stimulates and activates EGFR phosphorylation and MAPK activation in VSV-infected lung epithelial cells [38]. Thus, the results presented in this report clearly demonstrate that autocrine production of IL-8 plays a significant role in the proliferation of AIPC cells such as PC-3 and DU145, and enhance mitogen-stimulated cell cycle progression without any extrinsic source of IL-8.

We find that most of the CaP cell lines, that express androgen receptors with or without sensitivity to androgen-induced proliferation, do not express IL-8 under normal culture conditions. We tested this in LNCaP, LAPC-4, 22Rw21 and LNCaP-C4-2B (unpublished results). However, they do express IL-8 if stimulated by bacterial toxins [39] or under hypoxic conditions [40], thus demonstrating the plasticity of IL-8 expression in all CaP cells. We have shown previously that IL-8 level is increased in primary CaP tissues and is an independent predictor of biochemical (PSA relapse) recurrence [41], thus demonstrating its significance in primary tumor tissues (in prostate). The autocrine stimulation of IL-8 may be advantageous to proliferation, survival, motility and invasion, and resistance to cytotoxic drugs, when surviving in an ectopic environment, such as during seeding and growth in distant organs, such as bone and lungs. The ability to produce IL-8 in an autocrine fashion, with or without other survival and mitogenic factors, may be a critical determinant during initial survival and clonogenic proliferation in mitogen-poor environment or during total androgen-blockade. Indeed, Tso et al., [20] observed elevation of IL-8 as one of the key factors when they selected androgen-independent sub clones of LNCaP cells, an androgen-responsive cell line that does not secrete IL-8 [21].

Another significant finding of our study is that knockdown of endogenous IL-8 expression in AIPC cells reduces the NF-kB activity and phosphorylated-AKT level. In AIPC cells, AKT and NF-kB are constitutively activated and are known to exert significant effect on cell survival, resistance to anticancer drug-induced apoptosis and metastatic potential [41,42]. Whether constitutive activation of NF-kB is a cause of IL-8 production or constitutive production of IL-8 elevates NF-kB and AKT activity is not clear at present. However, at least in PC-3 and DU145 cells, it was recently elucidated that IL-8-CXCR2 interaction results in increased NF-kB activity during normal and stressed (drug or hypoxia) conditions [43]. Our results show that indeed, without external source of IL-8, constitutive activation of NF-kB is associated with expression of IL-8, as knockdown of IL-8 expression caused a significant inhibition of NF-kB activity and reduction in AKT phosphorylation (Fig. 4A and 4B). This corroborates our previous studies that suggested forced expression of IL-8 in androgen-sensitive cells causes constitutive activation of NF-kB (p65/rel) [21]. Constitutive IL-8 production activates AKT phosphorylation, but inhibition of AKT phosphorylation (by PI3Kinase inhibitor) did not prevent IL-8-mediated NF-kB activity, suggesting, IL-8 directly regulates NF-kB activation in AIPC cells, independent of AKT-mediated NF-kB activation [21].

We found that depletion of IL-8 causes a significant reduction in VEGF transcription and protein levels (Fig. 4C). This result was anticipated, as shown previously, CXCR2, the co-receptor of IL-8 stimulates VEGF transcription via G-protein mediated signaling [44]. Reductions in VEGF secretion and microvessel density have been reported earlier, in IL-8 reduced PC-3 tumors by IL-8 antisense transfection [22], and in other tumor systems [45].

We observed a significant decrease in invasive activity of PC-3 cells upon IL-8 depletion, as we had earlier observed, IL-8 up-regulation increasing the invasive potential of LNCaP and LAPC-4 cells, which are non-invasive *in vitro *[21]. Matrigel invasion involves both proteolytic activity by Type IV collagenase and chemotactic motility toward growth factors, present in the serum-containing medium at the bottom well of the chemotactic chamber. The role and mechanism of IL-8 as a chemokine has been well established, and so is an association between autocrine IL-8 production and increase in invasive enzymes, such as MMP-2 or MMP-9 [21,23]. Since the invasive activity was reduced by >48%, IL-8 appears to be a predominant chemokine in enabling chemo-invasive potential in AIPC cells.

We found increased spontaneous apoptosis in IL-8 depleted cells. This may be associated with simultaneous reduction of survival factors, cell cycle arrest, and increased levels of pro-apoptotic proteins, such as BAX. In addition, decrease in NF-kB activity may contribute a multitude of signaling networks with overall shift toward apoptosis. The NF-kB targeted gene includes inhibitor of apoptosis protein and the BCL-2 family of proteins [46-48]. Many of these pathways are involved in tumor growth, angiogenesis, metastasis, and resistance to chemotherapy in prostate and other tumors. BCL-2 expression is lower in localized prostate cancers compared with hormone refractory prostate cancer [49,50]. Over-expression of BCL-2 is one of many mechanisms that may enable prostate cancer cells to survive in an androgen-deprived environment [51,52]. The mechanism of over-expression of BCL-2 is largely undefined for AIPC. Previous study by Karl et al., [53] showed a link between BCL-2 and IL-8 in dermal microvessel endothelial cells, where over-expression of BCL-2 leads to increased secretion of IL-8, which in turn promoted angiogenesis. Wilson et al., [43] reported that some chemotherapy drugs, such as Oxaloplatin induce IL-8, this up-regulation leads to CXCR-2 mediated induction of BCL-2 and survivin expression. Furthermore, blocking of either CXCR2 or NF-kB activation down regulates BCL-2. Our results extend this observation to both anti-apoptotic proteins and pro-apoptotic proteins, BAX and BAD, with one difference. We did not use external IL-8 stimulation, but decreased the endogenous level that resulted in both transcriptional inhibition of BCL-2 and BCL-2 protein stability (Fig. 5A–D). Collectively, these finding suggest that IL-8 is the major regulator of chemoresistance in aggressive, AIPC cells and likely in patients with metastatic CaP. Indeed, IL-8 is prognostic marker for aggressive disease [41,54] and elevated levels of IL-8 in the plasma of patients with advanced (AIPC) disease have been reported [55].

Targeted therapy offers a unique opportunity to inhibit the activity of specific gene that is critical for growth and metastasis. It is significant to note that knockdown of IL-8 expression in PC-3 and DU145 cells with IL-8 siRNA significantly enhanced the chemotherapy responses as increased cytotoxicity. These observations might open a new opportunity to enhance the therapeutic efficacy of antitumor drugs: docetaxel, Staurosporine and rapamycin, in refractory tumors or in metastatic stage of AIPC. The combination of anti-IL8 and approved chemotherapy protocols may allow, not only reduction in the dose of the drugs, but also increased efficacy.

## Conclusion

We provide extensive evidence to demonstrate IL-8 mediated regulation of complex intracellular molecular signaling that leads to aggressive tumor cell behavior and increased survival during response to chemotherapy drug toxicity. We provide direct evidence for the control of anti-apoptotic protein expression by IL-8, both at the transcription and protein stability. The suppression of IL-8 using RNAi or specific cell permeable inhibitors of IL-8 or its receptors, may help sensitize AIPC to a wide variety of chemotherapeutic agents and might increase the survival of patients with end-stage disease.

## Materials and methods

### Reagents

Characterized fetal bovine serum was from Atlanta Biologicals (Atlanta, GA), Cell culture grade gentamicin, culture media, and transfection reagents were all from Gibco-Invitrogen (Carlsbad, CA). Both non-targeted, random sequence small interfering RNA (siRNA) and On-Target anti-IL-8siRNA were purchased from Dharmacon (Chicago, IL). The Smartpool On-Target siRNA were an equal mix of 4-siRNA species designed to hybridize and destroy human IL-8 mRNA (Gen Bank Accession No. NM_000584). These siRNAs were sequence verified to be specific to IL-8, thus eliminating the off-target effects. Dual-Glo luciferase assay kit was from Promega Corporation (Madison, WI). All reagents, other than primer sets, for real time, quantitative RT-PCR (Q-PCR) were from BioRad labs (Richmond, CA). All DNA primer sets for PCR and Q-RTPCR were custom designed and synthesized from Sigma-Genosys. Various primary and secondary antibodies were purchased from Cell signaling or Sigma-Aldrich, unless otherwise indicated.

### Cells and culture conditions

PC-3, LNCaP and Du145 cells were purchased from American Type Culture Collection, (ATCC, Manassas, VA) and were maintained *in vitro *in RPMI medium supplemented with 10% fetal bovine serum and gentamicin (2 μg/ml) and maintained at 5% CO_2_/37°C incubator.

### siRNA transfection and Real time PCR

The transient transfection of siRNA was performed with Dhramfect-2 transfection protocol (Dharmacon) with modifications. Briefly, the day before transfection, the 1 × 10^4 ^cells were plated in antibiotic free-RPMI medium with 10% FBS. Both on-target siRNA and Control siRNA were used at the same concentration in all experiments. Total RNA was isolated using RNAeasy mini kit (Qiagen Inc., Santa Clara, CA) following 48–72 hr transfection and used for reverse transcription real time PCR (qPCR) using the Bio-Rad iCycler iQ real time PCR system (Bio-Rad, Hercules, CA) with the gene specific primers. Primers sequences for IL-8-F 5'-ATGACTTCCAAGCTGGCCGTGCT-3'; IL-8-R 5'-TCTCAGCCCTCTTCAAAAACTTCT-3', VEGF F 5'-GCACCCATGGCAGAAGG-3'; VEGF-R5'-CTCGATTGGATGGCAGTAGCT-3', BCL-2-F 5'TGGGATGCCTTTGTGGAACT-3'; BCL-2-R GAGACAGCCAGGAGAAATCAAAC and glyceraldehyde 3-phosphate dehydrogenase (G3PDH) were G3PDH-F 5'TCCTCTGACTTCAACAGCGACAC-3'; G3PDH-R5'-CACCCTGTTGCTGTAGCCAAATTC-3'. The Q-PCR reaction was carried out using 2 μl of undiluted cDNA following the RT-reaction, and 0.225 μM of primer sets, and 2 × SYBR green master mix (BioRad). Regular PCR protocol was employed to time resolved PCR with an annealing temperature of 55°C for all primers annealed. Amplicon formation with each primer set was monitored with melt curve analysis. Gene expression was quantified relative to that of the housekeeping gene, cDNA for glyceraldehyde 3-phosphate dehydrogenase (G3PDH) as internal control. The threshold cycle (Ct) of each sample was determined by using SYBR green fluorescence of labled strands, and the relative level of expression (Fold difference, FD) was calculated as 1-(2^ΔCt^), where ΔCt = (test Ct - GAPDH Ct); data expressed as (1/FD) × 100, for easy to read integer numbers [21].

### Cell proliferation and drug sensitivity assay

Proliferation status of PC-3 and DU145 cultures, 48 h after siRNA transfection, were assessed using a colorimetric thiozolyl blue [3(4, 5-dimethylthiazol-2-yl)-2, 5-diphenyltetrazolium bromide (MTT) reduction assay or by direct cell counts, as described before [56]. Drug induced toxicity was determined following incubation with the indicated drug for 48 h with the control and siRNA transfected cultures. Cytotoxicity was normalized to that obtained with control siRNA transfected without drug-treated cultures.

### Determination of protein levels by immunoblotting (Western blotting)

Whole cell lysates prepared from treated cultures were fractionated on SDS-ployacrylamide gel electrophoresis and blotted on PVDF membranes (Millipore Inc., MA). Following blotting membrane was probed with antibodies specific for proteins of interest. Antibodies bound to target proteins were made visible by treating the membrane with enhanced chemoluminescence reaction using a kit (ECL ^plus ^kit, Amersham Pharmacia Biotech, Piscataway, NJ, USA) and exposing the membrane to X-ray film. Appropriate positive and negative control proteins, size markers and control cell lysates were loaded in parallel lanes to determine specificity of antibodies and minimize gel-to-gel variation. The blots were re-probed with antibody to β-actin to confirm equal loading of the solubilized samples. The intensity of specific protein bands were compared following digitization using a program (Kodak 1-D Gel analysis system, Kodak Corp, Rochester, NY).

### Quantitation of secreted proteins by ELISA

We assayed IL-8 and VEGF in the conditioned medium of various transfectants by enzyme immunoassays using commercial ELISA kits (Research Diagnostics Inc; R&D Systems) and the levels were normalized to cell number. Absolute levels of antigens, IL-8 and VEGF secreted by cultures were determined using the assay standards provided in the assay kits. Data are presented as Mean ± SEM from three separate experiments.

### Cell cycle phase-fractionation and estimation of cell cycles-phase fractions

We used flow cytometry to determine the DNA content of individual cells at 48 h following transfection with C-siRNA and IL-8 siRNA as described before [56]. Briefly, we harvested transfected or drug-treated cultures directly in a hypotonic solution containing Propidium iodide (50 μg/ml) and 0.04% NP-40 and the resulting suspension of nuclei was analyzed for DNA content using a flow cytometer (Beckman-Coulter Xcel, Miami, FL), in which 5 × 10^4 ^events were collected. The list mode data were regrouped into DNA histograms and individual cell-cycle phase-fraction was quantified using an analysis software (ModFit, Verity software Inc, Topsham, ME) [57].

### Determination of invasive activity

Invasive potential of transfected cells were determined by matrigel invasion assay as described before [21]. Briefly, cells were harvested 48 h after transfection with C-siRNA or IL-8siRNA using a hypotonic Cell stripper solution (Sigma-Aldrich) and suspended at 1 × 10^6 ^cells/ml in serum-free RPMI medium. The cell suspension (0.4 ml) was then placed on the top chamber of the Costar Transwell chamber plate previously coated with a basement membrane extract (Matrigel, Collaborative Research/BD Biosciences Inc, Bedford, MA). The lower compartment of Transwell was filled with 10% FBS in RPMI medium as chemo attractant or RPMI+ ITS medium as a control. Percent of invaded cells was estimated after 24 h incubation at 37°C in 5% CO_2_, using the MTT assay. Percent of cell population invading the Matrigel was calculated as a ratio of the optical density of cells in the top and bottom chambers [56]. Percent invaded cells = OD of the bottom wells/Total OD × 100. Experiment was repeated for two more times with independent transfections.

### Reporter assays

We assayed the activities of NF-kB using a reporter gene construct, as described before [21]. We plated 1 × 10^4 ^cells/well in 96-well plate and co-transfected with siRNA for IL-8 or C-siRNA, and 5 × NFKB-LUC (Stratagene/Agilent Technologies, San Diego, CA). Duplicate cultures treated identically, but co-transfected with TK Renilla plasmid (50 ng/ml) were used as internal control. Luminescence activity was measured using the Dual-Glo™ Luciferase Assay kit (Promega) as instructed. The activity of both the firefly and the Renilla Luciferase was determined in triplicate. Reporter activity was normalized to TK-Renilla luminescence and expressed in arbitrary units.

### Statistical analyses

All data reported in this report were generated using in vitro assays. The significance of the observation was estimated by Student's t-test, using data from at least three independent replicates, or by linear and non linear regression analysis, as indicated in each figure, except that of western blots, where the normalized band density was used to determine the significance. The observation was deemed significant if the probability of accepting null hypothesis is ≤ 0.05 (indicated by * in the Figures).

## Abbreviations

AIPC: Androgen-independent prostate cancer; AR: androgen receptor; IL-8: Interleukin 8; PSA: Prostate Specific Antigen; siRNA: Short interfering RNA; AP-1: Activating protein-1; NF-KB: Nuclear Factor Kappa B; VEGF: vascular endothelial growth factor.

## Competing interests

The authors declare that they have no competing interests.

## Authors' contributions

RKS and BLL performed experiments. Both authors were involved in the design and execution of the experiments, and writing the manuscript. BLL supervised the entire project in whose laboratory all experiments were preformed. Both authors read and approved the manuscript.
